# Supplementary material for: Cathepsin-facilitated invasion of BMI1-high hepatocellular carcinoma cells drives bile duct tumor thrombi formation
Source: Nat Commun. 2023 Nov 3;14:7033. doi: 10.1038/s41467-023-42930-y (PMC10624910; doi:10.1038/s41467-023-42930-y)
Supplement: Supplementary file 1 — Supplementary information [file 41467_2023_42930_MOESM1_ESM.pdf]

Supplementary Information

Cathepsin-facilitated invasion of BMI1-high hepatocellular carcinoma cells drives bile duct tumor thrombi formation

Lei-Bo Xu[1,2,3#], Yu-Fei Qin[1,2,3#], Liangping Su[1,2#], Cheng Huang[1,2], Qiuping Xu[1,2], Rui Zhang[1,2,3], Xiang-De Shi[1,2,3], Ruipu Sun[1,2], Jiali Chen[1,2], Zhixiao Song[1,2,3], Xue Jiang[1,2], Lihuan Shang[1,2], Xiao Gang[1,2,3], Xiangzhan Kong[1,2], Chao Liu[1,2,3*], Ping-Pui Wong[1,2*]

[1]Guangdong Provincial Key Laboratory of Malignant Tumor Epigenetics and Gene Regulation, Guangdong-Hong Kong Joint Laboratory for RNA medicine, Sun Yat-sen Memorial Hospital, State Key Laboratory of Oncology in South China, Sun Yat-sen University, Guangzhou, China 510120.

[2]Medical Research Center, Sun Yat-sen Memorial Hospital, Sun Yat-sen University, Guangzhou, China 510120.

[3]Guangzhou Key Laboratory of Precise Diagnosis and Treatment of Biliary Tract Cancer, Department of Biliary-Pancreatic Surgery, Sun Yat-sen Memorial Hospital, Sun Yat-sen University, Guangzhou, China 510120.

[#]These authors contributed equally to this MS.

***Correspondence:** liuchao3@mail.sysu.edu.cn, huangbp3@mail.sysu.edu.cn

Supplementary information file contains:

1. Supplementary Fig. 1-6
2. Supplementary Table 1-6

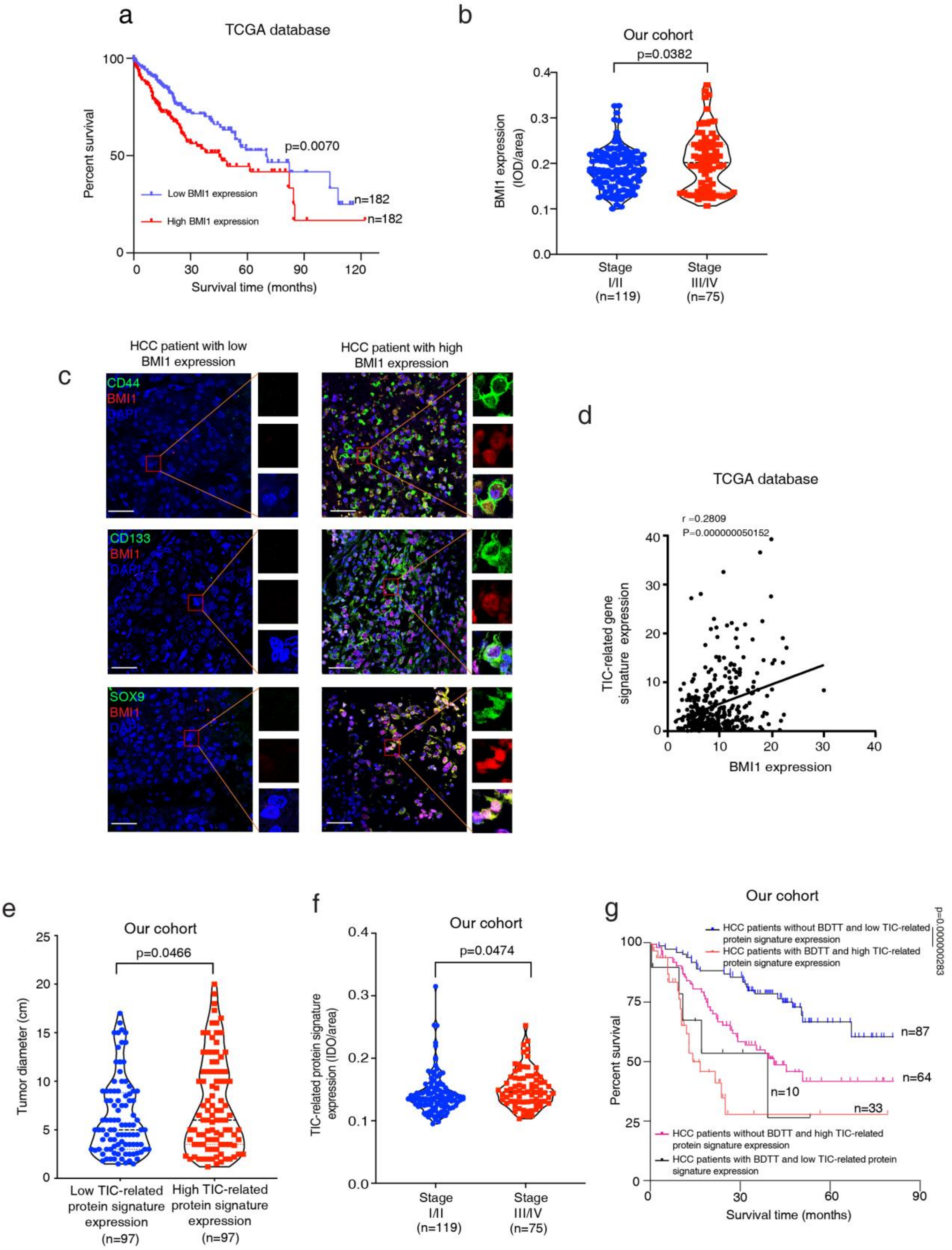

Supplementary Fig. 1. High expression of TIC-related gene/protein signature or BMI1 distinguishes HCC patients into those with poor prognosis. (**a**) Correlation between BMI1 expression and HCC patient overall survival in TCGA (phs000178.v11.p8) (n= 364 patients). (**b**) The relationship between BMI1 expression and cancer progression in HCC patients (n=194 patients, our cohort). (**c**) Representative immunofluorescent images of BMI1 and CD44/CD133/SOX9 stained

tumor sections from HCC patient with either high or low BMI1 expression are given. Magnified pictures are provided on the right-hand side. (**d**) Correlation study between TIC-related gene signature and BMI1 expression in HCC patients from TCGA (phs000178.v11.p8) (n= 364 patients). (**e**) High TIC-related gene signature expression corelated with enhanced tumor size in HCC patients (n=194 patients, our cohort). (**f**) The expression of TIC-related protein signature expression was higher in late staged HCC patients as compared to early staged patients (n=194 patients, our cohort). (**g**) Kaplan-Meier survival study of the relationship between HCC patients with BDTT and TIC-related protein signature expression and overall survival (n=194 patients, our cohort). Violin plots represent means ± S.E.M. (**b, e, f**) Unpaired two-tailed t-test. (**d**) Two-sided Pearson correlation test. (**a, g**) Log-rank (Mantel-Cox) test. Scale bars in (**c**) represent 100 μm.

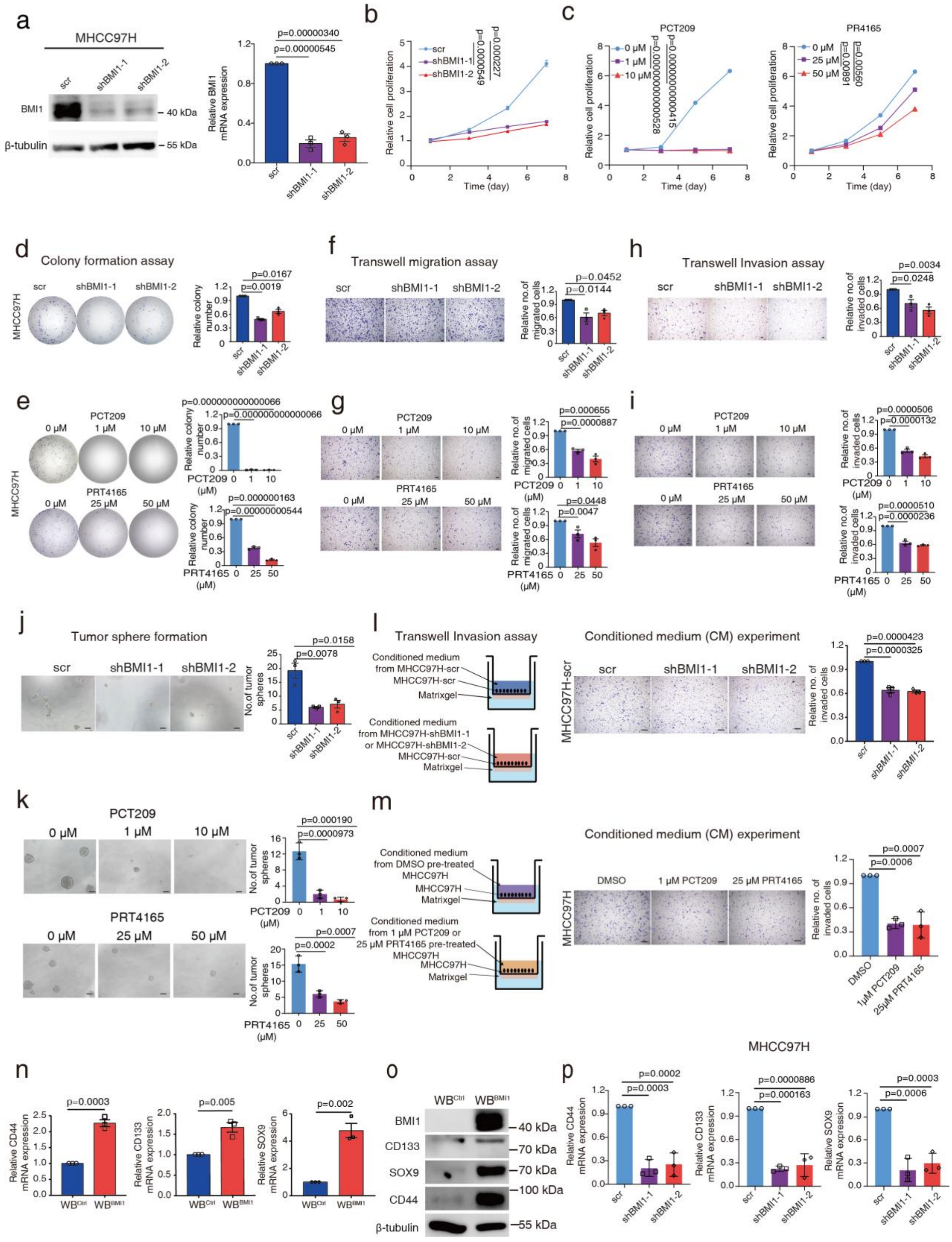

Supplementary Fig. 2. Depleting BMI1 reduces migration, invasion, colony formation and tumor sphere formation in HCC cells. (**a**) Western blot and RT-PCR analysis of the BMI1 expression in MHCC97H cells transfected with either scramble (MHCC97H-scr) or BMI1 targeting shRNA-1/-2 (MHCC97H-shBMI1-1/-2). Bar chart shows the relative BMI1 expression in MHCC97H-shBMI1-1/-2 after

normalization to MHCC97H-scr (n=3 independent experiments). Means ± S.E.M. (**b**) CCK8 proliferation assays of the BMI1 depleted MHCC97H cells and scramble transfected cells over time (n=5 independent experiments). (**c**) CCK8 proliferation assays of the MHCC97H cells after treated with DMSO or BMI1 inhibitors (i.e. PCT209 or PRT4165) at different doses over time (n=3 independent experiments). (**d**) Colony formation assay of MHCC97H-scr or MHCC97H-shBMI1-1/-2 cells (n=3 independent experiments). (**e**) Colony formation assay of MHCC97H cells after treated with DMSO, PCT209 or PRT4165 at indicated doses (n=3 independent experiments). (**f**) Transwell migration assays of MHCC97H-scr or MHCC97H-shBMI1-1/shBMI1-2 cells (n=3 independent experiments). (**g**) Transwell migration assay of MHCC97H cells after treated with DMSO, PCT209 or PRT4165 at indicated doses (n=3 independent experiments). (**h**) Tranwell invasion assays of MHCC97H-scr or MHCC97H-shBMI1-1/shBMI1-2 cells. (**i**) Transwell invasion assay of MHCC97H cells after treated with DMSO, PCT209 or PRT4165 at indicated doses (n=3 independent experiments). (**j**) Tumor sphere formation of MHCC97H-scr or MHCC97H-shBMI1-1/shBMI1-2 cells. (**k**) Tumor sphere formation of MHCC97H cells after treated with DMSO, PCT209 or PRT4165 at indicated doses (n=3 independent experiments). (**l**) Transwell invasion assay of MHCC97H-scr cells after exposed with conditioned medium (CM) from MHCC97H-scr/-shBMI1-1/-2 cells (n=3 independent experiments). (**m**) Transwell invasion assay of MHCC97H cells after exposed with CM from DMSO, PCT209 or PRT4165 pre-treated MHCC97H cells (n=3 independent experiments). (**n, o**) RT-PCR and western blot analysis of the expression of TIC-related markers in WB^{Ctrl} and WB^{BMI1} cells (n= 3 independent experiments). (**p**) RT-PCR analysis of the expression of CD44, CD133 and SOX9 in MHCC97H transfected with BMI1 targeting shRNA-1/-2 or scramble shRNA (n=3 independent experiments). Means ± S.E.M. (**a, d-m, p**) One-way ANOVA. (**b, c**) Two-way ANOVA test. (**n**) Unpaired two-tailed t-test. Scale bars in (**f, h, g-k, l, m**) represent 100 μm.

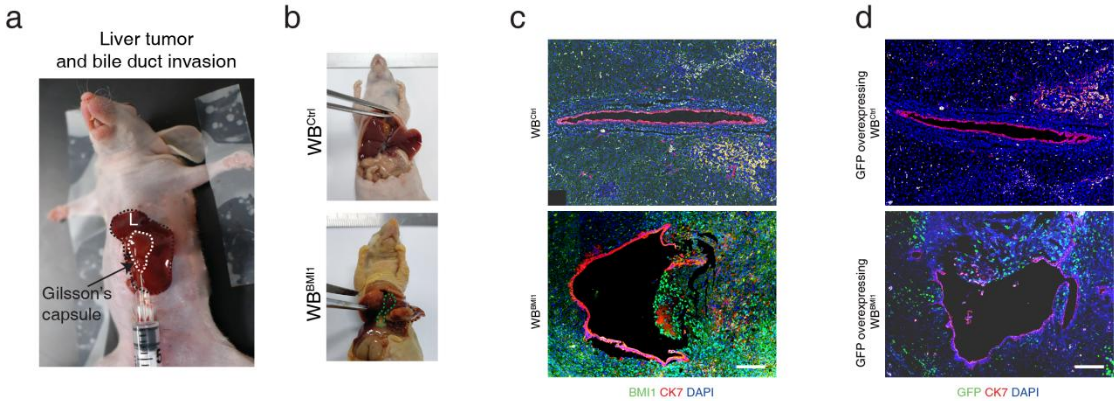

Supplementary Fig. 3. Visualization of tumor cell invasion and hepatic functions in nude mice implanted with WB^{Ctrl} or WB^{BMI1} cells. (**a**) Representative in situ gross image of orthotopic injection of WB^{Ctrl} or WB^{BMI1} cells into the Glisson's capsule of a nude mouse. L stands for liver. White dotted line indicated the position of Glisson's capsule and black dotted line for liver (n=5 mice per group). (**b**) Representative in situ gross pictures of orthotopic liver tumors 4 weeks after the injection from each group (n=5 mice per group). (**c**) Representative immunofluorescent images of BMI1 and CK7 stained tumor sections derived from WB^{Ctrl} or WB^{BMI1} implanted mice are given (n=5 mice per group). (**d**) Provided are representative immunofluorescent images capturing the GFP overexpressing BMI1 cells and CK7 antibody staining within tumor sections extracted from nude mice implanted with GFP labelled WB^{Ctrl} or WB^{BMI1} cells (n=5 mice per group). Scale bars in (**c, d**) represent 100 μm.

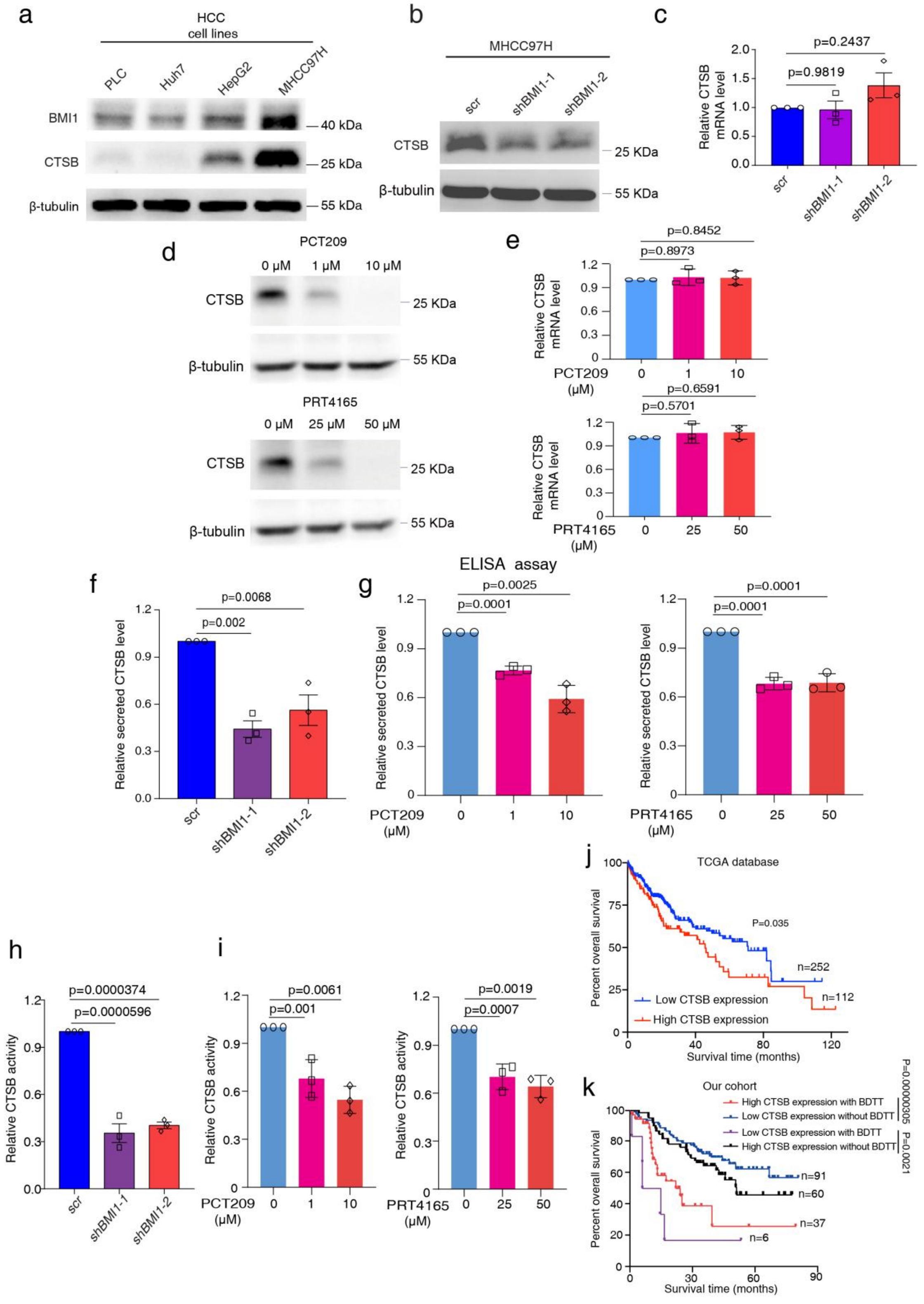

Supplementary Fig. 4. Depleting BMI1 reduces CTSB expression, enzymatic activity and secretion in HCC cells. (**a**) Western blot analysis of BMI1 and CTSB expression in a panel of human HCC cell lines (n=3 independent experiments). (**b, c**) Western blot and RT-PCR analysis of the BMI1 and CTSB expression in BMI1

depleted MHCC97H cells and scramble transfected cells (n=3 independent experiments). (**d, e**) Western blot and RT-PCR analysis of the CTSB expression in MHCC97H cells treated with either placebo or BMI1 inhibitors (i.e. PCT-209 or PRT4165) at indicated doses (n=3 independent experiments). Means ± S.E.M. (**f**) ELISA assay analysis of conditioned medium harvested from MHCC97H cells stably transfected with either scramble control shRNA or BMI1 targeting shRNA-1/-2. Bar chart represents means ± S.E.M. (**g**) ELISA assay analysis of conditioned medium harvested from MHCC97H after treated with either placebo or BMI1 inhibitors (i.e. PCT-209 or PRT4165) at indicated doses (n=3 independent experiments). (**h**) Measurement of the CTSB activity in conditioned medium derived from MHCC97H cells transfected with either scramble control shRNA or BMI1 targeting shRNA-1/-2. (**i**) Measurement of the CTSB activity in conditioned medium derived from MHCC97H cells after treated with either placebo or BMI1 inhibitors (i.e. PCT-209 or PRT4165) at indicated doses (n=3 independent experiments). (**j**) Correlation study between CTSB expression and overall survival in HCC patients from TCGA (n=364 patients). (**k**) Kaplan-Meier survival study of HCC patients with either high or low CTSB expression together with or without BDTT (n=194 patients, our cohort). (**c, e-i**) One-way ANOVA. (**j, k**) Log-rank (Mantel-Cox) test.

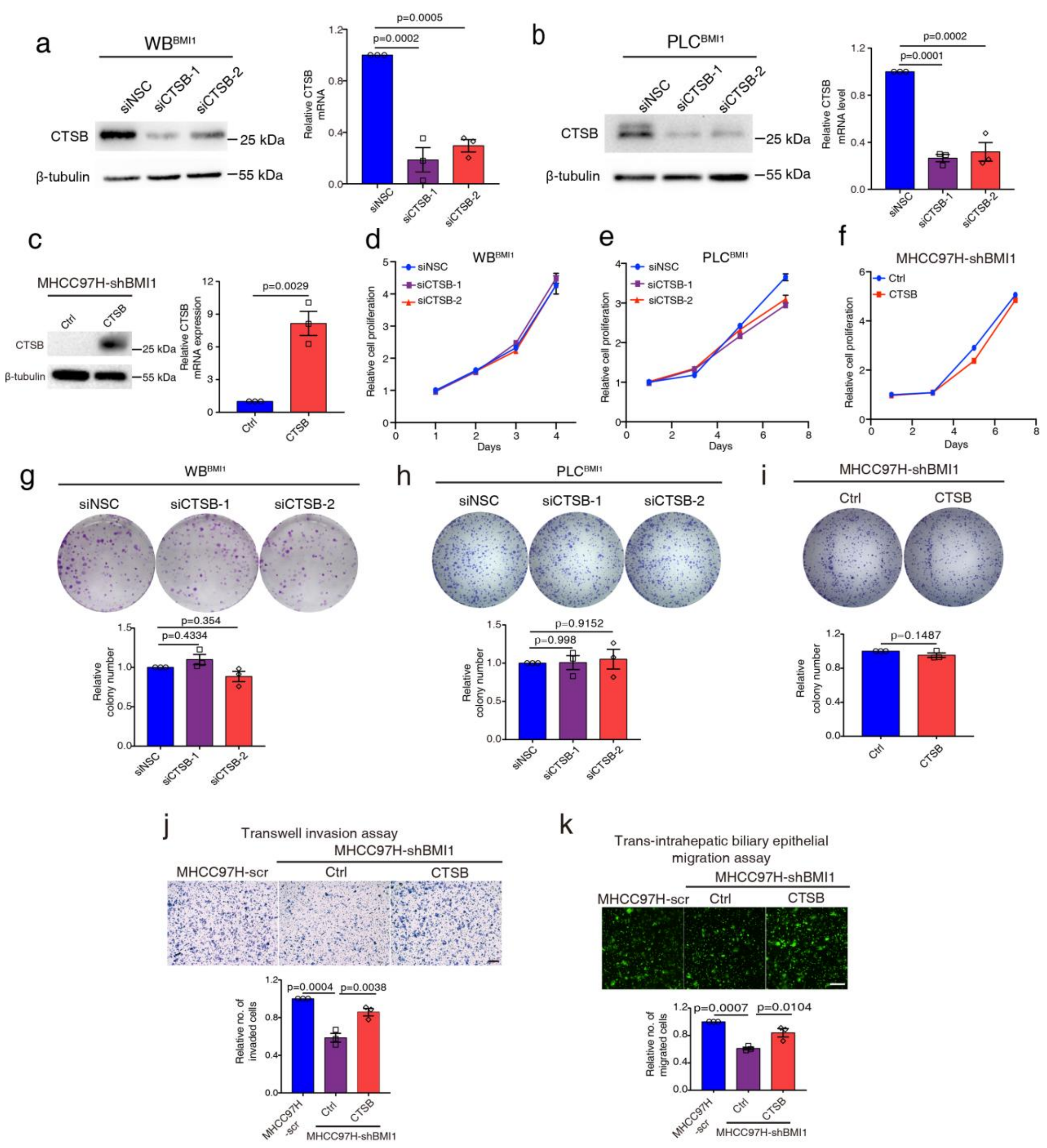

Supplementary Fig. 5. CTSB regulates the invasion and trans-intrahepatic biliary epithelial migration of stable BMI1 expressing cells without affecting their proliferation and colony formation. (**a, b**) Western blot and RT-PCR analysis of the CTSB expression in WB^{BMI1}/PLC^{BMI1} cells transfected with either CTSB targeting siRNA-1/-2 or non-silencing control siRNA (siNSC) (n=3 independent experiments). (**c**) Western blot and RT-PCR analysis of BMI1 stably depleted cells transfected with either empty vector or CTSB overexpression vector (n=3 independent experiments). (**d-f**) CCK8 proliferation of either WB^{BMI1}/PLC^{BMI1} cells transfected with CTSB targeting siRNA/siNSC or BMI1 depleted MHCC97H cells (MHCC97H-

shBMI1) transfected with empty vector/CTSB overexpression vector (n=4 independent experiments). (**g-i**) Representative images of crystal violet-stained colonies in each experimental group are given (n=3 independent experiments). Bar charts show the relative colony number in each experimental group. Means ± S.E.M. (**j**) Transwell invasion assay of MHCC97H-shBMI1 cells transfected with CTSB overexpression vector or empty vector (n=3 independent experiments). Representative images of crystal violet-stained invaded cells from each group are given. Scramble transfected MHCC97H cells were used as a control. (**k**) Trans-intrahepatic biliary epithelial migration assay of fluorescently labelled MHCC97H-shBMI1 cells transfected with CTSB overexpression vector or empty vector (n=3 independent experiments). Scramble transfected MHCC97H cells were used as a control. Representative images of fluorescently labelled migrated cells from each group are given. (**a-b, g, h, j, k**) One-way ANOVA**.** (**c, i**) Unpaired two-tailed t-test. (**d-f**) Two-way ANOVA.

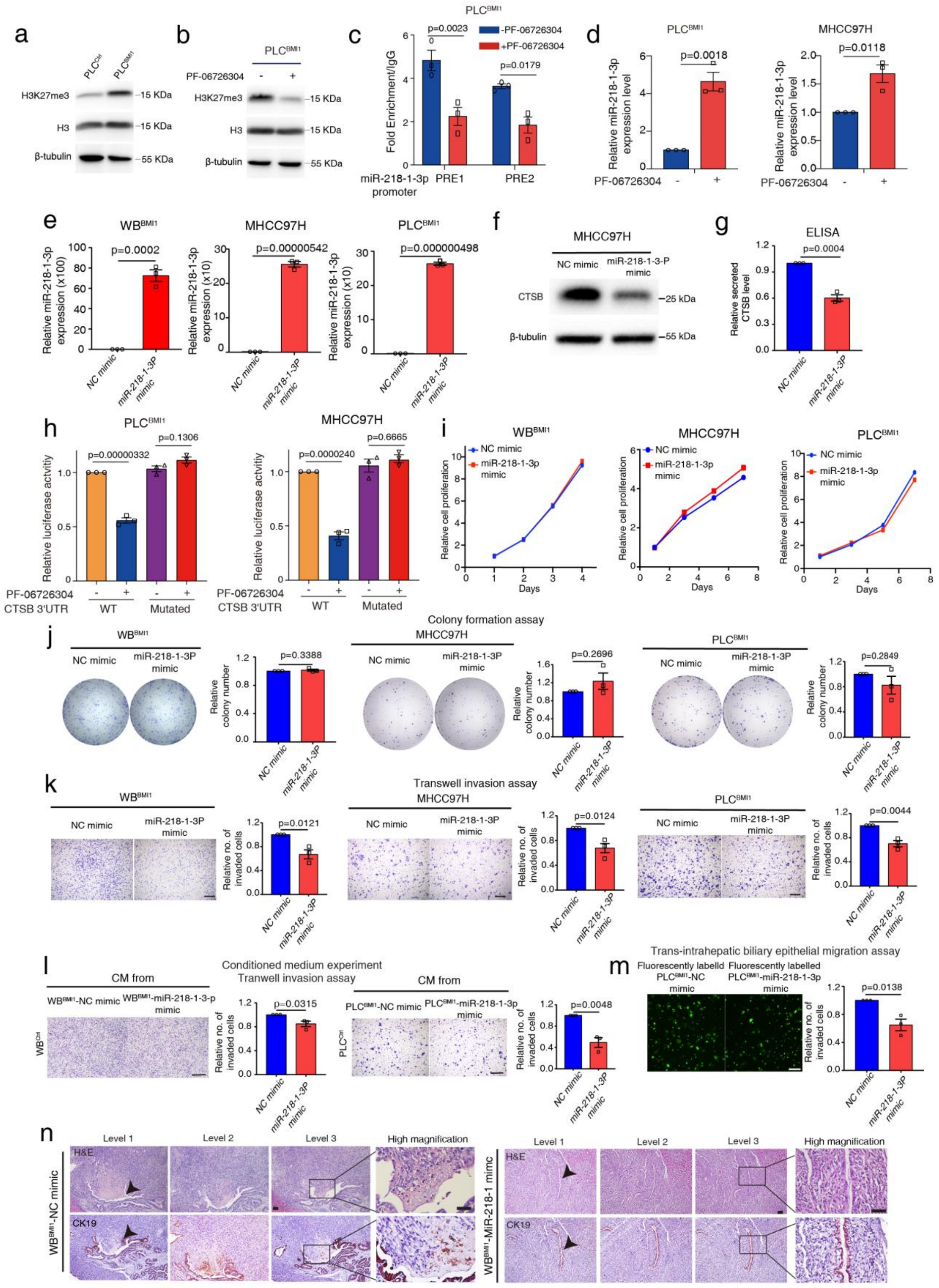

Supplementary Fig. 6. miR-218-1-3p overexpression prohibits bile duct tumor thrombosis in WB^{BMI1} orthotopic tumor bearing mice. (**a**) Western blot analysis of PLC^{Ctrl} and PLC^{BMI1} cells. β-tubulin was used as loading control. (**b**) Western blot analysis of PLC^{BMI1} cells treated with or without PF-06726034. (**c**) ChIP analysis of

H3K27me3 binding to the PRE1 and PRE2 sites in the miR-218-1-3p promoter in PLC^{BMI1} cells, with or without PF-06726034 treatment (n=3 independent experiments). (**d**) RT-PCR analysis of the level of miR-218-1-3p in PLC^{BMI1} and MHCC97H cells treated with or without PF-06726304. (**e**) RT-PCR analysis of the miR-218-1-3p expression in miR-218-1-3p mimic transfected WB^{BMI1}, PLC^{BMI1} and MHCC97H cells as compared to NC mimic transfected cells (n=3 independent experiments) (**f**) Western blot analysis for each experimental group (n=3 independent experiments). (**g**) ELISA analysis of the CTSB level in conditioned medium harvested from MHCC97H cells transfected with either miR-218-1-3p mimic or NC mimic (n=3 independent experiments). (**h**) Luciferase reporter assays of PLC^{BMI1} or MHCC97H cells after co-transfected with a luciferase reporter vector containing the sequence of either predicted wild type-3'-UTR (WT-CTSB) or mutated-3'-UTR region (Mut-CTSB) of CTSB and pLK reference plasmid with or without PF-06726034 treatment (n=3 independent experiments). (**i**) CCK8 proliferation growth curves of the indicated cell lines after stably transfected with miR-218-1-3p mimics or NC mimics (n=4 independent experiments for WB^{BMI} or PLC^{BMI1} cells; n=3 independent experiments for MHCC97H). (**j**) Colony formation assays for each experimental group (n=3 independent experiments). (**k**) Transwell invasion assays of WB^{BMI1}/MHCC97H/PLC^{BMI1}cells after stably transfected with miR-218-1-3p mimics or NC mimics (n=3 independent experiments). (**l**) Transwell invasion assays for each indicated experimental group (n=3 independent experiments). (**m**) Trans-intrahepatic biliary epithelial migration assay of fluorescently labelled WB^{BMI1}/PLC^{BMI1}cells after stably transfected with miR-218-1-3p mimics or NC mimics (n=3 independent experiments). (**n**) Representative pictures of H&E and CK19 immunohistochemical staining in serial tissue sections from each group are given. Arrow indicates the position of bile duct (n=10 mice per group). (**c, h**) One-way ANOVA. (**d, e, g, j-m**) Unpaired two-tailed t-test. (**i**) Two-way ANOVA. Scale bars in (**k-n**) represent 100 μm.

Supplementary Table 1. Characteristics of a cohort comprising 194 patients with hepatocellular carcinoma

Characteristics	No. of patients (%)
**Gender**	
Male	158 (81.44%)
Female	36 (18.56%)
**Age/years**	
Median	50
Range	23-80
**Drinking**	
No	160 (82.47%)
Yes	34 (17.53%)
**Cirrhosis**	
No	56 (28.87%)
Yes	137(70.62%)
**Tumorous number**	
Single	152 (78.35%)
Multiple	42 (21.65%)
**Tumor size**	
<5cm	112 (57.73%)
≥5cm	82 (42.27%)
**BDTT under the microscope**	
No	151 (77.84%)
Yes	43 (22.16%)
**AFP(µg/ml)**	
<20	69 (35.57%)
≥20	125 (64.43%)
**Vascular invasion**	
No	124 (63.92%)
Yes	70 (36.08%
**TNM stage**	
I-II	117(60.31%)
III-IV	77 (39.69%)

Supplementary Table 2. The list of RT-PCR primer sequences.

Gene name		Primer sequences
H-BMI1	Forward:	AATGTGAGGAAACTGTGGATG
	Reverse:	GTGACTCTGGGAGTGACAAGG
H-CTSB	Forward:	AGTCCCAAGAGTCGCAAGAAC
	Reverse:	GAATGCCTGTGCCAATAAAAG
H-GAPDH	Forward:	GCTGTGGGCAAGGTCATCC
	Reverse:	GCCTGCTTCACCACCTTCTT
R-BMI1	Forward:	CCAGGTGTAAATGTAGGCAAT
	Reverse:	CCAAAGGAGGAGGTGAATGAT
R-CTSB	Forward:	GACTCCTATCTGCCTCACTCC
	Reverse:	TACTGGCTGGTAGCAAACTCC
R-GAPDH	Forward:	TCTCTGCTCCTCCCTGTTC
	Reverse:	ACACCGACCTTCACCATCT
R-CD44	Forward:	CAAGTGCGAACCAGGACAGT
	Reverse:	AGATGCCAAGATGATGAGCC
R-CD133	Forward:	ACTTCCCTCAAGATTTGGTCA
	Reverse:	TGTTACAGCAACGGCACATAC
R-SOX9	Forward:	TGTATGTGGATGTGTGCGT
	Reverse:	CTGTCCGATGTCTCTCTGC
H-CD44	Forward:	CTGCCGCTTTGCAGGTGTA
	Reverse:	CATTGTGGGCAAGGTGCTATT
H-CD133	Forward:	AGTCGGAAACTGGCAGATAGC
	Reverse:	GGTAGTGTTGTACTGGGCCAAT
R-SOX9	Forward:	AGCGAACGCACATCAAGAC
	Reverse:	CTGTAGGCGATCTGTTGGGG

H stands for human

R stands for Rat

MiRNA name	Forward primer sequence	Reverse primer sequence
hsa-miR-548s	CGCAGATGGCCAAAACTG	Universal primer
hsa-miR-885-3p	GCAGCGGGGTGTAGTG	
hsa-miR-194-3p	CAGTGGGGCTGCTGT	
hsa-miR-4474-3p	GCAGTTGTGGCTGGTCA	
hsa-miR-1909-3p	GGGCCGGGTGCTC	
hsa-miR-4665-5p	GGGGGACGCGTGA	
hsa-miR-4269	GGCACAGACAGCCCT	
hsa-miR-4326	GTGTTCCTCTGTCTCCCA	
hsa-miR-30c-1-3p	GCTGGGAGAGGGTTGT	
hsa-miR-30c-2-3p	GCTGGGAGAAGGCTGT	
hsa-miR-3120-5p	CAGCCTGTCTGTGCCT	
hsa-miR-6086	AGGGAGGTTGGGAAGG	
hsa-miR-6715b-5p	ACAGGCACGACTGGT	
hsa-miR-4695-5p	GCAGGACAGGCAGAAGT	
hsa-miR-214-5p	GCCTGTCTACACTTGCTG	
hsa-miR-29b-2-5p	AGCTGGTTTCACATGGTG	
hsa-miR-4726-3p	ACCCAGGTTCCCTCTG	
hsa-miR-4525	GGGGGATGTGCATGCT	
hsa-miR-486-3p	GGGGCAGCTCAGTACAG	
hsa-miR-4421	GACCTGTCTGTGGAAAGG	
hsa-miR-4723-5p	GGGGGAGCCATGAGA	
hsa-miR-5698	GGGGGAGTGCAGTGA	
hsa-miR-5693	GGCAGTGGCTCTGAAATG	
hsa-miR-4516	CAGGGGAGAAGGGTCG	
hsa-miR-5196-3p	GTCATCCTCGTCTCCCT	
hsa-miR-4430	AGAGGCTGGAGTGAGC	
hsa-miR-4731-5p	GGGGGCCACATGAGT	
hsa-miR-30b-3p	AGCTGGGAGGTGGATG	
hsa-miR-5187-5p	CAGTGGGATGAGGGATTG	
hsa-miR-4747-5p	GGAAGGAGGCTTGGTCT	
hsa-miR-3652	GCGGCTGGAGGTGT	
hsa-miR-218-1-3p	GCAGATGGTTCCGTCAAG	
hsa-miR-486-5p	GCAGTCCTGTACTGAGCTG	
hsa-miR-154-5p	GCAGTAGGTTATCCGTGTTG	
hsa-miR-4438	AGCACAGGCTTAGAAAAGAC	
hsa-miR-455-5p	CAGTATGTGCCTTTGGACTAC	
hsa-miR-6132	AGCAGGGCTGGGG	
hsa-miR-138-5p	GCAGAGCTGGTGTTGTGA	
hsa-miR-5000-3p	GCAGTCAGGACACTTCTG	
hsa-miR-6131	GGCTGGTCAGATGGGA	
hsa-miR-4436a	GCAGGACAGGCAGAAG	

Supplementary Table 3. Liver blood function test in mice orthotopically liver-implanted with WB^{Ctrl} or WB^{BMI1} cells.

	Mouse no.	Total bilirubin (μmol/L)	Direct bilirubin (μmol/L)	γ-glutamyl -transferase(IU/L)
WB^{Ctrl}	1	0.5	0.7	0
	2	0.1	0.5	0
	3	0.3	0.5	0
	4	0.7	1.0	1.0
	5	0.6	1.4	0
WB^{BMI1}	1	208.7	270	36
	2	89.0	128.7	5.0
	3	0.5	1.0	17.0
	4	0.4	0.5	3.0
	5	1.1	1.7	3.0

Supplementary Table 4. Assessment of liver function in mice orthotopically spleen-implanted with WB^{Ctrl} or WB^{BMI1} cells.

Liver blood function test

Orthotopic splenic tumor model	Total bilirubin (μmol/L)	Direct bilirubin (μmol/L)	γ-glutamyltansferase (IU/L)
WB^{Ctrl}-Mouse 1	0.8	0.3	1
WB^{Ctrl}-Mouse 2	1.8	0.7	0
WB^{Ctrl}-Mouse 3	1	0.5	0
WB^{Ctrl}-Mouse 4	1	0.5	0
WB^{Ctrl}-Mouse 5	0.5	0.4	1
WB^{Ctrl}-Mouse 6	0.9	0.5	0
WB^{BMI1}-Mouse 1	151.2	121.9	16
WB^{BMI1}-Mouse 2	1.1	0.6	16
WB^{BMI1}-Mouse 3	0.9	0.3	9
WB^{BMI1}-Mouse 4	0.8	0.6	6
WB^{BMI1}-Mouse 5	0.9	0.3	5
WB^{BMI1}-Mouse 6	1	0.7	6

Supplementary Table 5. Liver blood function test in WB^{Ctrl} or WB^{BMI1} orthotopically liver-implanted mice, treated with placebo or CTSB inhibitor.

Liver blood function test

	Orthotopic liver model	Total bilirubin (μmol/L)	Direct bilirubin (μmol/L)	γ-glutamyltansferase (IU/L)
Placebo treatment group	WB^{BMI1}-Mouse 1	286.7	225.8	15
	WB^{BMI1}-Mouse 2	225.0	174.8	30
	WB^{BMI1}-Mouse 3	288.5	237.4	10
	WB^{BMI1}-Mouse 4	141.1	61.8	2
	WB^{BMI1}-Mouse 5	1	0.4	9
	WB^{BMI1}-Mouse 6	1.1	0.6	2
	WB^{BMI1}-Mouse 7	1.1	0.6	17
	WB^{BMI1}-Mouse 8	1.1	0.7	4
	WB^{BMI1}-Mouse 9	1.1	0.5	8
	WB^{BMI1}-Mouse 10	0.8	0.4	2
CTSB inhibitor treatment group	WB^{BMI1}-Mouse 1	0.5	0.5	1
	WB^{BMI1}-Mouse 2	1.2	0.5	5
	WB^{BMI1}-Mouse 3	1.2	0.8	1
	WB^{BMI1}-Mouse 4	1.3	0.5	7
	WB^{BMI1}-Mouse 5	0.7	0.5	0
	WB^{BMI1}-Mouse 6	1.0	0.5	1
	WB^{BMI1}-Mouse 7	0.7	0.4	4
	WB^{BMI1}-Mouse 8	1.0	0.5	1
	WB^{BMI1}-Mouse 9	1.0	0.5	7
	WB^{BMI1}-Mouse 10	0.4	0.1	0

Supplementary Table 6. Liver function blood test in mice orthotopically liver-implanted with WB^{BMI1} cells transfected with NC mimic or miR-218-1-3p mimic.

Liver blood function test

	Orthotopic liver model	Total bilirubin (μmol/L)	Direct bilirubin (μmol/L)	γ-glutamyltansferase (IU/L)
WB^{BMI1}-NC mimic group	Mouse 1	221.8	166.1	9
	Mouse 2	151.0	109.6	21
	Mouse 3	155.0	107.3	6
	Mouse 4	162.1	123.0	16
	Mouse 5	1	0.4	37
	Mouse 6	0.6	0.4	2
	Mouse 7	0.6	0.5	0
	Mouse 8	1	0.6	3
	Mouse 9	0.7	0.5	2
	Mouse 10	0.5	0.5	14
WB^{BMI1}-miR-218-1-3p mimic group	Mouse 1	71.0	53.3	7
	Mouse 2	0.5	0.5	0
	Mouse 3	1.0	0.2	11
	Mouse 4	0.4	0.4	5
	Mouse 5	0	0.2	1
	Mouse 6	0.8	0	3
	Mouse 7	0.1	0.3	1
	Mouse 8	0.7	0.1	5
	Mouse 9	0.6	0.6	4
	Mouse 10	0.4	0.3	0
