# Supplementary material for: Cathepsin-facilitated invasion of BMI1-high hepatocellular carcinoma cells drives bile duct tumor thrombi formation
Source: Nat Commun. 2023 Nov 3;14:7033. doi: 10.1038/s41467-023-42930-y (PMC10624910; doi:10.1038/s41467-023-42930-y)
Supplement: Supplementary file 3 — Reporting Summary [file 41467_2023_42930_MOESM3_ESM.pdf]

nature portfolio

Corresponding author(s): Ping-Pui Wong

Last updated by author(s): Oct 23, 2023

Reporting Summary

Nature Portfolio wishes to improve the reproducibility of the work that we publish. This form provides structure for consistency and transparency in reporting. For further information on Nature Portfolio policies, see our Editorial Policies and the Editorial Policy Checklist.

Statistics

For all statistical analyses, confirm that the following items are present in the figure legend, table legend, main text, or Methods section.

n/a	Confirmed	
☐	☒	The exact sample size (*n*) for each experimental group/condition, given as a discrete number and unit of measurement
☐	☒	A statement on whether measurements were taken from distinct samples or whether the same sample was measured repeatedly
☐	☒	The statistical test(s) used AND whether they are one- or two-sided<br>*Only common tests should be described solely by name; describe more complex techniques in the Methods section.*
☐	☒	A description of all covariates tested
☐	☒	A description of any assumptions or corrections, such as tests of normality and adjustment for multiple comparisons
☐	☒	A full description of the statistical parameters including central tendency (e.g. means) or other basic estimates (e.g. regression coefficient) AND variation (e.g. standard deviation) or associated estimates of uncertainty (e.g. confidence intervals)
☐	☒	For null hypothesis testing, the test statistic (e.g. *F*, *t*, *r*) with confidence intervals, effect sizes, degrees of freedom and *P* value noted<br>*Give P values as exact values whenever suitable.*
☒	☐	For Bayesian analysis, information on the choice of priors and Markov chain Monte Carlo settings
☐	☒	For hierarchical and complex designs, identification of the appropriate level for tests and full reporting of outcomes
☐	☒	Estimates of effect sizes (e.g. Cohen's *d*, Pearson's *r*), indicating how they were calculated

Our web collection on statistics for biologists contains articles on many of the points above.

Software and code

Policy information about availability of computer code

Data collection	Images were obtained by using either a confocal microscope (Zeiss LSM 800) or a light microscope (Nikon NI-U).<br>RT-PCR data were obtained by Roche LightCycler 480 II.<br>Optical density data were obtained by TECAN Spark10M.<br>Proteomics data were obtained by Orbitrap Exploris 480 System.<br>Western Blot data were obtained by Mini Chemi 610.
Data analysis	Carl Zeiss ZEN Version 2<br>NIS-Elements Version F 4.60.00<br>Image Pro Plus Version 6.0<br>Graphpad Prism Version 8.0.1<br>LightCycler 480 Version 1.5.1<br>Proteome Discoverer software suite (Version 2.3, ThermoFisher Scientific)<br>Xcalibur software Version 4.0

For manuscripts utilizing custom algorithms or software that are central to the research but not yet described in published literature, software must be made available to editors and reviewers. We strongly encourage code deposition in a community repository (e.g. GitHub). See the Nature Portfolio guidelines for submitting code & software for further information.

Data

Policy information about availability of data

All manuscripts must include a data availability statement. This statement should provide the following information, where applicable:

- Accession codes, unique identifiers, or web links for publicly available datasets
- A description of any restrictions on data availability
- For clinical datasets or third party data, please ensure that the statement adheres to our policy

The publicly available mRNA sequencing and clinicopathological data of patients with HCC used in this study are accessible in the Cancer Genome Atlas Program (TCGA) database under accession code phs000178.v11.p830 (https://www.ncbi.nlm.nih.gov/projects/gap/cgi-bin/study.cgi?study_id=phs000178.v11.p8) (PMID: 28622513). The use of these publicly available data from HCC was also consulted on the website (http://kmplot.com/analysis/index.php?p=service&cancer=liver_rnaseq) according to the web tool's instruction (PMID: 30662724), under the specific product name: KM Plotter-Liver Cancer. The proteomics data generated in the study has been deposited tin the Proteome X consortium via the PRIDE partner repository under accession code PXD037074, https://www.iprox.cn//page/project.html?id=IPX0005126000).

Human research participants

Policy information about studies involving human research participants and Sex and Gender in Research.

Reporting on sex and gender	Previous research has shown that gender is not linked to the occurrence of BDTT. Consequently, gender was not taken into account as a variable in this study. The clinicopathological characteristics of HCC patients included in this investigation, such as sex/gender, quantity, age, alcohol consumption, presence of cirrhosis, tumor count, tumor size, microscopic analysis of BDTT, AFP levels, vascular invasion, and TNM stage, are elaborated in Supplementary Table 1.
Population characteristics	For our patient cohort study, we included individuals with hepatocellular carcinoma (HCC) who underwent surgical resection at Sun Yat-sen Memorial Hospital of Sun Yat-sen University between 2012 and 2018. During this period, a total of 1791 cases of HCC patients underwent liver cancer resection surgery, and among them, 53 cases were identified as having combined bile duct cancer thrombi (BDTT). The inclusion criteria for this study were as follows: 1. Pathological diagnosis of liver cancer; 2. R0 resection; 3. Availability of complete clinical data and follow-up information; 4. Access to relevant specimens. Exclusion criteria comprised: 1. Prior preoperative radiotherapy or chemotherapy; 2. Concurrent presence of other tumors. The diagnostic criteria for HCC patients with combined bile duct cancer thrombi required the observation of tumor foci within the bile duct lumen under the microscope, along with a pathological diagnosis of liver cancer combined with bile duct cancer thrombi. Based on the aforementioned selection criteria, we included 151 cases of patients without bile duct cancer thrombi and 43 cases of patients with combined bile duct cancer thrombi in this study. Detailed clinicopathological characteristics, including gender, quantity, age, alcohol consumption, cirrhosis status, tumorous number count, tumor size, microscopic examination of BDTT, AFP levels, vascular invasion, and TNM stage, are provided in Supplementary Table 1.
Recruitment	Clinical samples with comprehensive clinical data were procured from Sun Yat-sen Memorial Hospital in Guangzhou, China. Each individual's clinical specimens were obtained at the time of surgery.
Ethics oversight	This study was approved by the ethnical review committee of Sun Yat-sen Memorial Hospital in Guangzhou, China.

Note that full information on the approval of the study protocol must also be provided in the manuscript.

Field-specific reporting

Please select the one below that is the best fit for your research. If you are not sure, read the appropriate sections before making your selection.

☒ Life sciences ☐ Behavioural & social sciences ☐ Ecological, evolutionary & environmental sciences

For a reference copy of the document with all sections, see nature.com/documents/nr-reporting-summary-flat.pdf

Life sciences study design

All studies must disclose on these points even when the disclosure is negative.

Sample size	In this study, a formal statistical method for sample size calculation was not employed. The sample size of the TCGA database (TCGA-LIHC;phs000178.v11.p830 (https://www.ncbi.nlm.nih.gov/projects/gap/cgi-bin/study.cgi?study_id=phs000178.v11.p8))was determined by the number of tumor samples analyzed through RNA sequencing, along with the availability of corresponding clinicopathological data (PMID: 28622513; PMID:30662724). Similarly, for our patient cohorts, the sample size was not statistically determined prior to collection. Details regarding the sample size and the number of independent experiments conducted are provided in the figure legend. Statistical analysis was performed using three or more independent samples/experiments.
Data exclusions	No data were excluded from the analyses
Replication	Every experiment was independently repeated a minimum of three times. The precise number of replicates for each experiment is explicitly

	stated in both the methods section and the figure legend.
Randomization	In animal experiments, mice of similar ages and weights were randomly assigned to different experimental groups, with each group receiving the specified treatments. For experiments not involving animals, samples/cells were randomly allocated to different experimental groups before treatment or observation.
Blinding	The researchers responsible for conducting the animal experiments were unblinded, as they were required to administer the specific treatments to the mice. However, blinding was upheld during the subsequent data analysis. While conducting microscopy and gathering data through objective instruments, the researchers were aware of the group allocations for the raw data, as this information was crucial at that stage. Nevertheless, they maintained blinding during the subsequent data analysis.

Reporting for specific materials, systems and methods

We require information from authors about some types of materials, experimental systems and methods used in many studies. Here, indicate whether each material, system or method listed is relevant to your study. If you are not sure if a list item applies to your research, read the appropriate section before selecting a response.

Materials & experimental systems

n/a	Involved in the study	
☐	☒	Antibodies
☐	☒	Eukaryotic cell lines
☒	☐	Palaeontology and archaeology
☐	☒	Animals and other organisms
☒	☐	Clinical data
☒	☐	Dual use research of concern

Methods

n/a	Involved in the study	
☒	☐	ChIP-seq
☒	☐	Flow cytometry
☒	☐	MRI-based neuroimaging

Antibodies

Antibodies used	rabbit mAb against human BMI1 (CST, 6964), 1:1000<br>rabbit mAb against human/ mouse Cathepsin B (CST, 31718), 1:1000<br>rabbit mAb against human/mouse Sox9 (Abcam, ab185966), 1:1000<br>rabbit mAb against human CD44 (CST, 37259), 1:1000<br>rabbit mAb against human/mouse CD133 (Abcam, ab19898), 1:1000<br>rabbit mAb against human/mouse β-tubulin (Abcam, ab179513), 1:1000<br>rabbit mAb against human/mouse Cytokeratin 19(Abcam, ab52525), 1:500<br>rabbit mAb against human/mouse Cytokeratin 7(Abcam, ab181598), 1:6000<br>goat mAb against rabbit horseradish peroxidase (HRP)-conjugated antibody (CST, 7074S) , 1:1000
Validation	The antibody validation information corresponding to above are as following:<br>https://www.cellsignal.cn/products/primary-antibodies/bmi1-d20b7-xp-rabbit-mab/6964?site-search-type=Products&N=4294956287&Ntt=bmi1&fromPage=plp<br>https://www.cellsignal.cn/products/primary-antibodies/cathepsin-b-d1c7y-xp-rabbit-mab/31718?site-search-type=Products&N=4294956287&Ntt=ctsb&fromPage=plp<br>https://www.abcam.cn/sox9-antibody-epr14335-78-ab185966.html<br>https://www.cellsignal.cn/products/primary-antibodies/cd44-e7k2y-xp-rabbit-mab/37259?site-search-type=Products&N=4294956287&Ntt=cd44&fromPage=plp<br>https://www.abcam.cn/cd133-antibody-stem-cell-marker-ab19898.html<br>https://www.abcam.cn/beta-tubulin-antibody-epr16774-ab179513.html<br>https://www.abcam.cn/cytokeratin-19-antibody-ep1580y-cytoskeleton-marker-ab52625.html<br>https://www.abcam.cn/cytokeratin-7-antibody-epr17078-cytoskeleton-marker-ab181598.html<br>https://www.cellsignal.cn/products/secondary-antibodies/anti-rabbit-igg-hrp-linked-antibody/7074?site-search-type=Products&N=4294956287&Ntt=7074&fromPage=plp&_requestid=7146255

Eukaryotic cell lines

Policy information about cell lines and Sex and Gender in Research

Cell line source(s)	Rat liver progenitor cell WB-F344 was purchased from the Cell Bank of the Chinese Academy of Science (Cat no.#CTCC-400-0377, Shanghai, China). Human intrahepatic biliary epithelial cells (HIBEpiC) were purchased from ScienCell (Cat no.#5100). Human HCC cell lines (i.e. PLC (Cat no.# CTCC-003-0017), Huh7 (Cat no.#CTCC-003-0019) and HepG2 (Cat no.#CTCC-001-0014), MHCC97H (Cat no.#CTCC-400-0192)) were obtained from the Cell Bank of the Chinese Academy of Sciences (Shanghai, China).
Authentication	Cell lines were used as provided commercially and no additional identification was performed.
Mycoplasma contamination	No mycoplasma contamination was detected during the study.

Commonly misidentified lines (See ICLAC register)	No commonly misidentified lines were used in the project.

Animals and other research organisms

Policy information about studies involving animals; ARRIVE guidelines recommended for reporting animal research, and Sex and Gender in Research

Laboratory animals	Male nude mice aged 4-6 weeks were obtained from Slac Laboratory Animal Co. Ltd. (Hunan, China). After their acquisition, the mice were allowed a one-week acclimatization period at the animal facility of Sun Yat-sen University. During the experiments, they were maintained under specific conditions, including an ambient temperature of 22-24 °C, humidity control between 40%-70%, and a 12-hour light/dark cycle. They were provided with access to sufficient food and water.
Wild animals	No wild animals were used in this study.
Reporting on sex	Male mice were utilized in this study. Past research has established that gender is unrelated to the incidence of BDTT. Therefore, gender was not considered a variable in this study.
Field-collected samples	No field-collected samples were used in this study.
Ethics oversight	All animal procedures carried out in this study were approved by the institutional animal care and use committee of Sun Yat-sen university

Note that full information on the approval of the study protocol must also be provided in the manuscript.
